# Making it stick: use of active learning strategies in continuing medical education

**DOI:** 10.1186/s12909-020-02447-0

**Published:** 2021-01-11

**Authors:** Brenda A. Bucklin, Nancy L. Asdigian, Joy L. Hawkins, Ulrich Klein

**Affiliations:** 1 University of Colorado School of Medicine, 12631 East 17th Avenue | Mail Stop 8202, Aurora, CO 80045 USA; 2 Colorado School of Public Health, University of Colorado Anschutz Medical Campus, Aurora, CO USA; 3 University of Colorado School of Dental Medicine, Aurora, CO USA

**Keywords:** Active learning, Continuing medical education, Educational strategies, Medical education

## Abstract

**Background:**

Despite the known benefits of active learning (AL), the predominate educational format in higher education is the lecture. The reasons for slow adaptation of AL in medical education are not well understood. The purpose of this survey was to determine knowledge, usage, attitudes, and barriers to AL use in academic Continuing Medical Education (CME).

**Method:**

A 20-item questionnaire was developed and sent with a link to an online questionnaire to the Society of Academic Continuing Medical Education (SACME) listserv of ~ 350 professionals representing academic medical centers, teaching hospitals, and medical specialty societies in the United States (U.S.) and Canada. Responses were collected with SurveyMonkey® from October–November, 2019. Data were analyzed using SPSS®.

**Results:**

Responses from 146 SACME members in 91 CME units yielded a ~ 42% survey response rate. Many respondents reported their self-perceived knowledge of AL as high. Advanced training (e.g., certificate, Master of Education degree) was positively correlated with AL knowledge. AL methods were reportedly used in half of the CME activities in the majority (80%) of institutions. Higher levels of self-perceived knowledge were correlated with an increased percentage of AL-related CME activities. Commonly perceived barriers to use of AL were presenters’ lack of familiarity and a need for more time-consuming preparation.

**Conclusions:**

More efforts are needed to increase innovation and incorporate evidence-based AL strategies in medical education, especially to foster learner engagement, critical thinking, and problem-solving ability.

**Supplementary Information:**

The online version contains supplementary material available at 10.1186/s12909-020-02447-0.

## Introduction

Teacher-centered lectures continue to be largely utilized in higher education overall. However, leaders in medical education have challenged the lecture format because active learning (AL) strategies have been shown to promote better retention and application of new knowledge than listening to passive, information-only imparting lectures [1–3]. Fostering curiosity and discovery while critically evaluating and discovering viable ways to address gaps in professional practice, change practice, and improve patient outcome are necessary for life-long learning in medicine. While biomedical information grows at an exponential rate and email inboxes are flooded with learning opportunities, learners seek relevant, efficient, and meaningful educational forums.

Active learning describes a broad range of learning activities (e.g., flipped classroom, think-pair-share, turn and talk, bulleted breaks during lecture) that require a learner to “construct”, understand, and comprehend the knowledge derived from their educational experience “while simultaneously improving knowledge gain and recall abilities” [4]. Although AL strategies were introduced nearly 40 years ago, reports of use in medical education are limited, but the benefits of AL have been well described. Increased content knowledge, critical thinking, problem-solving abilities, and positive attitudes towards learning have been noted when inquiry-based cooperative learning was compared to traditional lecture-based learning formats [5]. In addition, development of graduate capabilities such as critical and creative thinking, problem-solving, adaptability, communication and interpersonal skills were observed when AL strategies were used in graduate education [6].

Freeman et al. concluded, “If the experiments analyzed here had been conducted as randomized controlled trials of medical interventions, they may have been stopped for benefit—meaning that enrolling patients in the control condition might be discontinued because the treatment being tested was clearly more beneficial” [7].

Although AL is effective, there are opportunities to learn more about knowledge, usage, attitudes and barriers to use of AL methods. By improving content delivery, knowledge retention and self-directed learning are encouraged.

A knowledge gap about use of AL learning strategies extends across all areas of medical education, including continuing medical education (CME) [8]. Our research efforts emerged from the results of a 2018 survey of academic CME unit leaders in the U.S. and Canada suggesting that the lecture is the most common educational delivery method in CME [9]. An earlier report in the CME literature highlighted the importance of using learner-centric AL formats to engage the learner with the teacher and audience to increase self-awareness and critical thinking [3]. As in other learner groups, use of AL methods in CME has been shown to reinforce content and promote changes in physician practice with improvement in patient outcome [10]. Given the importance of these methods in changing practice, there is a substantial gap in the literature about the reasons surrounding the limited use of these newer learning strategies in CME. By understanding more about knowledge, usage, attitudes and barriers to use of AL methods, the results of this research will provide CME programs with an opportunity to learn and adapt so that CME units can improve the learning environment, using their unique position within medical education to influence physicians’ practice. By creating greater awareness of AL methods across the educational continuum, there is an opportunity to engage learners by making lessons “stickier” (more comprehensible and memorable) and utilizing “lecture halls without lecture” [1]. CME is just one area on this continuum of medical education, however similar questions and survey tools can be used to extend our knowledge and awareness of the benefits of AL in other areas of medical education.

## Method

Potential survey respondents were leaders of CME units and members of Society of Academic Continuing Medical Education (SACME) at 144 U.S., 17 Canadian, and other CME medical education units (e.g., American College of Surgeons, American Academy of Pediatrics). SACME is the academic society for continuing medical and interprofessional education that advances continuing education through scholarship by promoting and advancing high-value healthcare. Respondents were identified through the SACME Communications Committee listserv.

While the 2018 Harrison Survey provided the AL construct for the study and reported that the lecture format is the most commonly used learning format in CME, the survey results provide a widesweeping “snapshot of the current structure and function of CME/CPD units at medical schools in the United States and Canada.” [9] and did not specifically address AL strategies. Consequently, the literature was searched extensively for publications about AL, but there have been no surveys published that represent the construct in its entirety. Questions were subsequently developed for this de novo survey based on 6 content areas that were identified through another comprehensive review of existing literature about AL use in medical education and higher education in general: 1) demographics; 2) knowledge; 3) usage; 4) attitudes about; 5) barriers to use; and 6) potential strategies to address those barriers [4, 7, 9, 11]. AMEE Guide No. 87 [12], a guideline publication outlining best practices for survey reporting [13], and an expert in survey design and administration from the University of Colorado School of Public Health influenced item development. Subsequently, 20 draft items consisting of multiple choice, matrix, yes/no, and free-text questions were pretested by content experts (including 15 members of the SACME communications and scholarship committees) as well as the expert in survey design. After this initial round of survey item revision, cognitive interviews were conducted with 7 randomly selected SACME members. This was followed by a final round of survey item revision, instrument formatting, and refinement. Altogether, pretesting, cognitive interviews, and expert reviews followed by further revision helped to establish content and response process validity.

The Active Learning Survey in CME was conducted under an IRB-approved protocol (#19–1274) through the University of Colorado School of Medicine. An introductory email announcing the upcoming survey was distributed to ~ 350 SACME members by the Content Manager of SACME. Survey invitations were then sent by this individual directing individual members to a specific SurveyMonkey® link (Active Learning Survey in CME). This email explained the purpose and objectives of the survey. They were informed that they were voluntarily consenting to participate in a research study when completing the survey. No compensation or incentives were provided. The 20-question survey took approximately 10 min to complete. Four email reminders were sent to non-respondents during the 6-week study period in October–November, 2019.

Demographic data were collected. Respondents were asked about their perceived level of knowledge about AL methods with a four-point Likert scale (very knowledgeable, mostly knowledgeable, somewhat knowledgeable, not at all knowledgeable). Respondents were also asked what type of, if any training they had received in AL methods (e.g., certificate or Master of Education). Their beliefs and the extent of agreement or disagreement (strongly agree, agree, neither agree or disagree, disagree, or strongly disagree) with attitudinal statements about AL were also assessed in the survey. CME unit leaders were asked about the use of AL compared to lecture in CME activities at their institution. AL strategies were defined for participants in a linked online Teaching Practice Terminology Tip Sheet. If respondents reported that AL strategies were used in their unit, they were prompted to identify which type of strategy was used. Survey respondents were also asked to indicate the degree to which various factors made it more difficult to use AL in their CME unit. This was followed by a question about how helpful various methods would be in facilitating the use of AL strategies in their unit. Respondents were also invited to complete open-ended questions about best practices for use of AL at their institution. A link to the Association of American Medical Colleges (AAMC) Faculty Roster 2018 was provided for respondents to identify the size of faculty at their institution.

Non-responders were identified by review of medical school IP addresses not represented in the data set. Ten non-responders were contacted by phone to participate in a brief scripted follow-up telephone survey focused on a subset of key survey questions, including demographics, use of AL in their CME unit, and barriers to usage. This telephone survey was conducted by the principal investigator to identify potential differences and extent of non-response bias between responders and non-responders. Sample size for this survey was limited by the fixed number of academic CME units in the U.S. and Canada.

Data from de-identified survey responses were collected electronically with SurveyMonkey® and analyzed using IBM® SPSS® Statistics 26.0 (publication date: April 9, 2019; https://www.ibm.com/analytics/spss-statistics-software) software platform. The primary outcomes analyzed were the overall knowledge, attitudes about, use, and barriers to use of AL in CME. All demographic data were summarized using descriptive statistics (means, standard deviations, frequencies, percentages). Spearman’s correlations were used to assess statistical associations between: 1) advanced training in AL (e.g., certificate, Master, or Doctor of Education degree) and self-perceived knowledge of AL; 2) self-perceived knowledge of AL and percentage of CME activities in their institution using AL; and 3) advanced training in AL (e.g., Certificate, Master’s, or Doctor of Education degree) and percentage of CME activities using AL in their institution*.* Internal consistency and reliability of the survey scores were assessed with Cronbach’s or Krippendorf’s alpha coefficients, which were calculated for each subconstruct measured.

## Results

We were able to collect 146 responses. This represents ~ 42% of SACME unit leaders from 93 U.S. and Canadian medical schools as well as other medical specialty societies (e.g., American College of Surgeons, American Academy of Pediatrics). Since not all respondents answered all questions due to skip-logic survey methodology, not all data sets are complete. The number of full-time faculty in medical schools represented ranged from 150 to 8000 with a mean of 1406 full-time faculty members per responding institution. Seventy percent of the responding CME unit leaders were based in a medical school. More than 50% were CME Unit Directors and Assistant/Associate Deans of CME. Just over half of participating unit leaders had been in their positions for more than 6 years. Other demographics are described in Table 1.

**Table 1:** Respondent and Continuing Medical Education Unit Demographics for Continuing Medical Education Unit Leaders in the U.S. and Canada, 2019

Age	
25-34	2.0% (2)
35-44	15.0% (16)
45-54	21.0% (23)
55-64	35.0% (38)
65+	18.0% (20)
I prefer not to answer	9.0% (10)
Missing	*n*=37
Position of CME Leader	
CME Unit Director	36.4% (37)
Asst/Assoc Dean of CME	17.6% (18)
CME Program Manager	7.8% (8)
Other Education Leader	4.9% (5)
CME Unit Administrator	2.9% (3)
CME Unit Coordinator	2.9% (3)
Other	27.5% (28)
Missing	*n*=44
Gender	
Female	64.0% (64)
Male	29.0% (29)
I prefer not to answer	7.0% (7)
Missing	*n*=46
Medical School-based CME unit	
Yes	65.2% (60)
No	34.8% (32)
Missing	*n*=54
ACCME Accreditation of unit	
Yes, with commendation	58.7% (54)
Yes, without commendation	28.3% (26)
No	13.0% (12)
Missing	*n*=54

*CME* Continuing Medical Education

*ACCME* Accreditation Council of Continuing Medical Education

*Asst/Assoc* Assistant/Associate

*U.S.* United States

Data are represented as % (n) of respondents

**Fig. 1:**
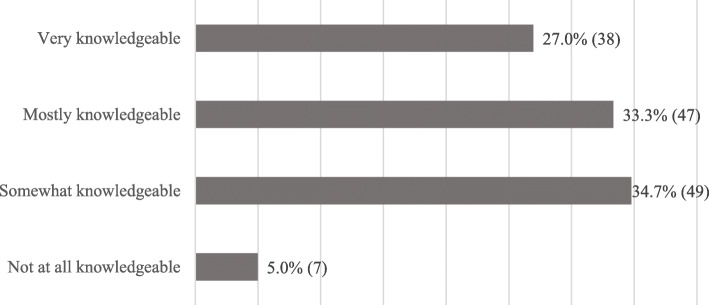
Respondents’ Self-perceived Knowledge about Active Learning in Continuing Medical Education Units in the U.S. and Canada, 2019. Data are presented as % (n) of respondents. Missing *n* = 5. U.S. = United States

Sixty percent of the survey participants described themselves as very or mostly knowledgeable about AL (see Fig. 1). Approximately 80% of respondents had participated in national continuing education programs (e.g., annual conferences) that included information about AL methods. In addition, ~ 50% had participated in a continuing education program at their institution and/or had engaged in self-study that included information about AL. Fewer respondents had participated in on-line courses. More than one-third of the survey respondents had completed a certificate, Master, or Doctor of Education degree that included information about AL methods. Spearman’s correlation revealed that advanced training was positively correlated with self-perceived knowledge of AL (Spearman’s correlation = 0.23 at *P* = .01).

More than 80% of respondents reported that AL methods were used in less than half of CME activities at their institutions. Simulation, audience response polling, case-based teaching, small group activities, and panel discussions were used most commonly (> 90%). Active learning strategies used in CME units, as reported by CME unit leaders are outlined in Table 2. Respondents also noted other examples of AL methods including gaming, group reflection with coaching between sessions, and role playing. While advanced training was not significantly correlated with an increased percentage of CME activities using AL (Spearman’s correlation = 0.14 at *P* = .06), higher levels of self-perceived knowledge were correlated with an increased percentage of CME activities using AL in respondents’ institutions (Spearman’s correlation = 0.16 at *P* = .04).

**Table 2:** Active Learning Strategies Currently Used in 2019 by Continuing Medical Education Units in the U.S. and Canada Using Descriptive Statistics and Krippendorff’s alpha from Survey Questions

Active Learning Methods Utilized	Yes	No	I don’t know
Case-based discussion	99.2% (117)	0.0% (0)	0.8% (1)
Panel discussion	99.2% (117)	0.8% (1)	0.0% (0)
Simulation exercises	96.4% (108)	2.7% (3)	0.9% (1)
Audience Response Polling	94.1% (111)	3.4% (4)	2.5% (3)
Small group discussion	94.1% (111)	5.1% (6)	0.8% (1)
Large group discussion	87.1% (101)	6.9% (8)	6.0% (7)
Self-reflection exercises	73.3% (85)	17.2% (20)	9.5% (11)
Think-Pair-Share	66.1% (76)	22.6% (26)	11.3% (13)
Flipped classroom	63.6% (75)	29.7% (35)	6.8% (8)
Peer observation and feedback	61.7% (66)	29.0% (31)	9.3% (10)
Turn and Talk	58.9% (66)	21.4% (24)	19.6% (22)
Pause procedures during lecture	34.0% (36)	31.1% (33)	34.9% (37)
Bulleted breaks during lecture	17.2% (20)	49.1% (57)	33.6% (39)
One-minute paper	7.0% (8)	67.0% (77)	26.1% (30)
Others used *n* = 17	**Krippendorff’s alpha**	**95% CI**	
		Lower Bound	Upper Bound
	0.27	0.26	0.27

*U.S.* United States

*95% CI* 95% Confidence Interval

Data are represented as % (n) of respondents

More than 90% of respondents agreed or strongly agreed that AL combines engagement with reflection. They also agreed that AL requires cooperation of the instructor to engage learners by working together to increase knowledge and recall (see Table 3).

**Table 3:** Attitudes and Perceptions about Use of Active Learning in Continuing Medical Education Unit Leaders in the U.S. and Canada During 2019 Using Descriptive Statistics and Cronbach’s alpha from Survey Questions

Attitudes about Active Learning	Strongly Agree/Agree	Neither Agree nor Disagree	Disagree/ Strongly Disagree
AL combines engagement and observation with reflection	94.2% (114)	3.3% (4)	2.5% (3)
The learner is engaged so that both knowledge gained and recall are increased in AL	93.4% (113)	5.8% (7)	0.8% (1)
Both the instructor and the learners work cooperatively in AL	90.9% (110)	5.8% (7)	3.3% (4)
With AL, instructors are more concerned with eliciting reflective thoughts that apply knowledge to practice than merely conveying facts	75.2% (91)	17.4% (21)	7.4% (9)
AL changes the teacher-learner relationship to a learner-learner relationship	68.6% (83)	26.4% (32)	5.0% (6)
Lectures (passive learning) are difficult to adapt to AL methods ^a^	22.3% (27)	14.9% (18)	62.8% (76)
	**Cronbach’s alpha**	**95% CI**	
		Lower Bound	Upper Bound
	0.74	0.63	0.79

^a^ item excluded from Cronbach’s alpha analysis

Mean (SD) 4.19 (0.25)

*AL* Active Learning

*U.S.* United States

*95% CI* 95% Confidence Interval

Data are represented as % (n) of respondents

Most commonly perceived barriers to use of AL are lack of familiarity of faculty/presenters with AL methods in addition to increased preparation and planning for AL. Other barriers are described in Table 4. Respondents suggested that guidelines for use of AL strategies, faculty development programs to include training in AL, and coaching course directors to discuss AL methods, are examples of how CME unit leaders could increase AL content in CME activities. Other resources that were noted by respondents in assisting them in developing AL strategies in their institutions are presented in Table 5.

**Table 4:** Perceived Barriers for Using Active Learning in Continuing Medical Education Units in the U.S. and Canada During 2019 Using Descriptive Statistics and Cronbach’s alpha from Survey Questions

Barriers to Active Learning	Strongly Agree/Agree	Neither Agree nor Disagree	Disagree/ Strongly Disagree
Peers and colleagues are not familiar with AL teaching practices ^a^	100.0% (1)	0.0% (0)	0.0% (0)
AL requires more preparation and planning as it is an unfamiliar teaching format for many presenters	93.9% (93)	1.0% (1)	5.1% (5)
Many lecturers / presenters are unfamiliar with AL teaching methods	82.9% (92)	12.6% (14)	4.5% (5)
Most presenters “grew-up” and studied in a predominately lecture-based system and it worked for them; so, they don’t see a need to change	63.6% (63)	20.2% (20)	16.2% (16)
Interaction with the audience may expose knowledge gaps of the lecturer/presenter	53.0% (53)	28.0% (28)	19.0% (19)
The culture of my institution is lecture-based	48.0% (48)	28.0% (28)	24.0% (24)
At my institution, there is a lack of administrative / secretarial support for AL	45.5% (50)	28.2% (31)	26.4% (29)
Course attendees are unfamiliar with AL	41.7% (5)	25.0% (3)	33.3% (4)
Learners dislike AL because it requires their participation	34.0% (35)	25.2% (26)	40.8% (42)
AL is incompatible with larger audiences	27.0% (30)	15.3% (17)	57.7% (64)
My institution does not offer faculty development opportunities for AL teaching methods	26.9% (29)	18.5% (20)	54.6% (59)
At my institution, AL is not considered a useful teaching tool in CME	15.9% (18)	22.1% (25)	61.9% (70)
AL is an inefficient way of teaching because increased interaction with the audience is time-consuming	12.7% (14)	14.5% (16)	72.7% (80)
Lectures require less preparation or planning by instructors ^a^	0.0% (0)	100.0% (1)	0.0% (0)
Other barriers *n* = 7	**Cronbach’s alpha**	**95% Confidence Interval**	
		Lower Bound	Upper Bound
	0.82	0.76	0.87

^a^ items excluded from Cronbach’s alpha analysis

mean (SD) 3.09 (0.66)

*CME* Continuing Medical Education

*AL* Active Learning

*U.S.* United States

*95% CI* 95% Confidence Interval

Data are represented as % (n) of respondents

**Table 5:** Suggested Resources for Increasing Use of Active Learning Methods in Continuing Medical Education Units in the U.S. and Canada in 2019 Using Descriptive Statistics and Cronbach’s Alpha from Survey Questions

Resources for Increasing the Use of AL	Very helpful/ moderately helpful	Somwhat helpful	A little helpful/ Not at all helpful
AL-related faculty development materials	89.2% (99)	9.9% (11)	0.9% (1)
Assistance from educational strategists	85.6% (95)	8.1% (9)	6.3% (7)
Institutional recognition for AL teaching / curriculum development	83.8% (93)	13.5% (33)	2.7% (8)
Institution-wide training sessions on AL	67.6% (75)	22.5% (25)	9.9% (11)
Best-practice publications about AL	63.1% (70)	29.7% (33)	7.2% (8)
Other resources n = 3	**Cronbach’s alpha**	**95% CI**	
		Lower Bound	Upper Bound
	0.82	0.75	0.87

mean (SD) 4.09 (0.24)

*AL* Active Learning

*U.S.* United States

*95% CI* 95% Confidence Interval

Data are represented as % (n) of respondents

Score validity and reliability of the survey instrument were assessed by applying Messick’s five sources of validity evidence [14]. While the new instrument was being developed, we collected evidence for content validity from an exhaustive literature review and consultation with content experts for question review and revision. Response process validity was also examined by testing the questions by experts and reviewers before distributing the survey. Since construct validity reflects the extent to which a measure ‘behaves’ the way that the underlying construct should behave in relation to other constructs, Spearman’s correlations were used to establish this type of validity. Score reliability of the survey instrument is reported in Tables 2, 3, 4 and 5.

When addressing potential non-response bias with telephone interviews, we found results similar to the online survey. Most of the 10 non-respondents that were questioned by phone were CME Unit Directors or Program Managers and 45–64 years of age. Less than 50% of these telephone interviewees used AL in CME activities at their institutions. Barriers included a lack of familiarity with AL methods, more time-consuming planning and preparation, and a lecture-based culture at their institutions.

## Discussion

Increasing awareness of AL and its lack of use as well as extending the use of this AL survey instrument and methodology to other areas of medical education were important goals for publication of this study. However, the primary goal of this survey was to extend the results of the 2018 Harrison survey and address unanswered questions about the knowledge, usage, attitudes, and barriers to usage of AL strategies in academic CME units in U.S. and Canada. The results suggest that many CME unit leaders are well-trained and have education in AL as well as positive attitudes towards the use of AL methods, but the change from the lecture format to implementation of AL methods has been minimal. While the mission of the Accreditation Council for Continuing Medical Education (ACCME) is to “assume and advance quality learning for healthcare professionals that drives improvements in patient care” [15], the translation of knowledge into practice that improves patient outcome is complex [16]. Although much has been written about the quality of health care delivered in the U.S. [17, 18], the challenge for CME is to deliver impactful medical knowledge in a way that providers are able to retain and engage with the delivered knowledge in order to stay current and improve patient care.

In general, faculty members have not kept pace with pedagogical advancements and knowledge of medical education delivery methods [2]. Little is known about the actual training and knowledge of CME unit leaders in AL methods. In our study, we found that many of the CME unit leaders have both formal and informal training that included information about AL. More than half of the respondents consider themselves knowledgeable about AL methods. Learners in these institutions may have an edge since engagement and translation of the knowledge facilitates changes in practice and improved patient outcomes [10].

While the practice of medicine has changed dramatically during the last half century and knowledge and information have proliferated over the last several decades [19], medical classroom teaching has not changed substantially and is still driven by the lecture format [9]. Our study confirms that change in teaching methods comes slowly. Despite the fact that most of the respondents in our survey described themselves as knowledgeable about AL methods, 80% reported that AL is used in less than half of the CME activities at their institutions. When AL strategies were used, audience response polling, simulation, and group discussions were the most common. We asked survey respondents about the source of their responses to determine the accuracy of their reporting. They indicated that their answers were mostly based on factual CME unit data and frequent attendance at lectures, courses, or conferences at their institutions, validating the accuracy of our study findings. Taken together, the positive correlation of advanced training and self-perceived knowledge with use of AL suggest that many CME unit leaders are trained to use and facilitate these teaching methods in their institutions.

There are many AL strategies for instructors to consider when they design courses, but negative attitudes and pedagogical barriers must be overcome [4]. When exploring these potential attitudes of CME unit leaders, our survey demonstrates that survey participants recognize to a large degree the importance of learner engagement to increase knowledge and recall. They also agree that instructors who use AL are more concerned with facilitating reflection and working cooperatively with the learner. As a result, instead of merely conveying facts to the learner, knowledge can be applied in practice. While more than 60% of survey participants indicate that lectures are not difficult to adapt to AL methods, instructors at their institutions, nevertheless, do not incorporate AL methods to a large degree into their CME activities. Our study confirms that the benefits of AL are recognized but practical implementation still lags behind in many institutions.

While respondents have knowledge, positive attitudes, and perceptions about AL, we still needed to explore barriers to AL and describe resources needed to overcome these barriers to better understand the challenges of CME unit leaders in their institutions. In STEM disciplines, barriers to use of AL methods are described as “complex” [11] and include the triad of lack of time, training, and incentives to implement educational change. In medical education, however, barriers to use of AL have not previously been described. In our study, we found similar barriers to those identified by the STEM disciplines and the need for additional resources for preparation, planning, and faculty development. Other barriers include a lack of administrative support and change-averse cultures at institutions. Similar to the STEM disciplines, assistance from educational designers, institutional recognition, as well as additional training and faculty development in AL was found to be useful for increasing the use of AL in CME.

The strengths of this study are the methods used to develop the survey. Questions used to develop the construct, AL, were developed through an in-depth literature review of AL. Survey development was also guideded by Messick’s five sources of validity evidence [14]. Although content and response process validity are strong because of use of extensive pretesting and cognitive interviewing of the study population prior to dissemination, the lack of established criterion validity is a limitation of the study and will be an important next step in this area of research. Buy-in from SACME facilitated survey distribution to ensure that all members of the society had an opportunity to respond to the survey. Following survey distribution and data collection, we evaluated reliability of the survey instrument. Although one of our subconstructs, ‘Active learning strategies currently used in CME units’ had lower internal consistency reliability related to the yes/no question type and distribution of the data, the Likert scale survey questions demonstrated high internal consistency reliability as confirmed by Cronbach’s alpha.

Limitations of the study include low response rates as observed with on-line surveys. Reasons for this include potential incorrect email addresses, survey fatigue of the study population, and membership changes within SACME, whose membership is dynamic. The varying number of SACME listserv members, directly impacts the denominator. There were also more female compared to male respondents. Studies suggest that distribution of online survey responses by gender is variable and dependent upon the survey population and characteristics of that population. Drawing conclusions about these gender differences would be purely speculative. Although voluntary responses improve the quality of the responses, non-response bias must also be considered. Response rates to questionnaires have been declining over time regardless if the queries are Web-based or when other data collection technologies are used [20]. Multiple reasons for non-response have been recognized and despite utilization of recommended techniques and processes (e.g., prenotification of participants, publicizing the questionnaire) [12, 13, 20], we report an ~ 42% response rate. Our goal of performing follow up telephone surveys in non-respondents was to determine if respondents to the questionnaire were different from those who did not respond in terms of demographic or attitudinal variables. Since non-response bias also increases the likelihood of differences in the group of respondents compared to non-respondents, we followed up with telephone surveys of 10 randomly selected nonrespondents to validate the results of selected questions. Results of these telephone surveys were similar to online survey results, indicating a small effect of non-response bias on our results. Although some institutions may have been represented by several respondents, the anonymous nature of the survey and heterogeneity in responses limits this concern. The non-validated nature of self-reported items such as training, knowledge, and use of AL represent other recognized limitations. Finally, the use of AL cannot make up for poor event content, even if AL is used as the teaching method.

This study is the first to expand one conclusion of the 2018 Harrison survey by describing the knowledge, usage, attitudes, and barriers to implementation of AL strategies in academic CME units in the U.S. and Canada. Our results suggest that increased levels of self-perceived knowledge but not advanced training are associated with increased use of AL. Despite CME unit leaders’ training, education, and positive attitude toward AL, the use of AL is infrequent in many CME units, often resulting from limited resources such as faculty development and institutional recognition for AL teaching. Future directions include extending the survey and its methodology to other areas of medical education such as undergraduate and graduate medical education, incorporating qualitative data (e.g., in-depth narrative data) to gain better depth of understanding of the AL construct, as well as follow-up with similar surveys done in tandem with other aspects of the Harrison survey to trend the data. We would welcome collaboration by the AAMC and SACME to increase awareness of AL and the considerations of its use and implrmentation.

## Conclusion

By improving dissemination of information about evidence-based AL strategies in medical education, the results of this study can be used to increase educational innovation that fosters learner engagement, critical thinking, and problem-solving ability.

## Supplementary Information

**Additional file 1.**

## Data Availability

Data used and/or analysed for the study are available from the corresponding author after a request.

## Abbreviations

AAMC: Association of American Medical Colleges
ACCME: Accreditation Council of Continuing Medical Education
AL: Active Learning
CME: Continuing Medical Education
CME/PD: Continuing Medical Education/Professional Development
SACME: Society for Academic Continuing Medical Education
STEM: Science, Technology, Engineering, Mathematics
