# Supplementary material for: Making it stick: use of active learning strategies in continuing medical education
Source: BMC Med Educ. 2021 Jan 11;21:44. doi: 10.1186/s12909-020-02447-0 (PMC7798232; doi:10.1186/s12909-020-02447-0)
Supplement: Supplementary file 1 — Additional file 1. [file 12909_2020_2447_MOESM1_ESM.pdf]

# Active Learning in Continuing Medical Education

## Knowledge of Active Learning

**What is Active Learning? Compared to a lecture format, active learning (AL)** ***is defined as any instructional method that engages learners in the learning process. In short, AL requires learners to participate in meaningful learning activities, think about and analyze what they are doing and learning*****. Examples of AL methods include: think-pair share, pause procedure during a lecture, or case-based discussions.**

**For explanations of the official AL terms, please see** **Teaching Practice Terminology Tip Sheet** **for details.**

**Note: for all questions you will need to complete all answers before moving to the next question or page.**

**The following abbreviations will be used throughout the questionnaire:**
**AL = Active Learning; CE = Continuing Education; CME = Continuing Medical Education.**

**The first set of questions will ask you about your knowledge of active learning.**

* 1. Have you completed or participated in any of the following formal or informal training and/or program activities that included instruction about AL?

| | Yes | No |
|---|---|---|
| A Certificate, Master's, or Doctor of Education degree that included information about AL methods | ◯ | ◯ |
| CE program in your Institution that included information about AL format | ◯ | ◯ |
| National CE programs (e.g., annual conferences) that included information about AL | ◯ | ◯ |
| On-line courses about AL | ◯ | ◯ |
| CE credits awarded by Medical Education journals (e.g., writing or peer review) | ◯ | ◯ |
| Self-study about AL | ◯ | ◯ |
| Other degree program | ◯ | ◯ |

If you answered "yes" to "self-study" or "other degree program", please describe.

* 2. How knowledgeable do you perceive yourself about AL as an educational method?

◯ Very knowledgeable

◯ Mostly knowledgeable

◯ Somewhat knowledgeable

◯ Not at all knowledgeable

## Active Learning in Continuing Medical Education

### Use of Active Learning

**The following questions are about your CME program's use of active learning methods.**

* 3. How knowledgeable are you about whether AL is used in CME courses or programs certified by your CME unit?

- Very knowledgeable
- Mostly knowledgeable
- Somewhat knowledgeable
- Not at all knowledgeable

## Active Learning in Continuing Medical Education

### Use of Active Learning

* 4. Based on your knowledge of these CME activities, please estimate the percentage of CME activities that are delivered by AL method.

- 0%
- 1-25%
- 26-50%
- 51-75%
- 76-100%

* 5. Your estimate for question 4 about percentage of CME activities using AL is based on...

- Actual data review
- Your overall perception (i.e., "gut feel")
- Frequent attendance at lectures/courses/conferences
- "Good-faith" estimate


## Active Learning in Continuing Medical Education

### Use of Active Learning

* 6. Please tell us which of the following AL methods are used in CME activities certified by your CME unit. For explanations of the official AL terms used below, please see Teaching Practice Terminology Tip Sheet for details.

| | Yes | No | I don't know |
|---|---|---|---|
| Flipped classroom | ◯ | ◯ | ◯ |
| Think-Pair-Share | ◯ | ◯ | ◯ |
| Turn and Talk | ◯ | ◯ | ◯ |
| Audience Response Polling | ◯ | ◯ | ◯ |
| Pause procedures during lecture | ◯ | ◯ | ◯ |
| Bulleted breaks during lecture | ◯ | ◯ | ◯ |
| One-minute paper | ◯ | ◯ | ◯ |
| Peer observation and feedback | ◯ | ◯ | ◯ |
| Simulation exercises | ◯ | ◯ | ◯ |
| Self-reflection exercises | ◯ | ◯ | ◯ |
| Case-based discussion | ◯ | ◯ | ◯ |
| Panel discussion | ◯ | ◯ | ◯ |
| Small group discussion | ◯ | ◯ | ◯ |
| Large group discussion | ◯ | ◯ | ◯ |

What others are used? (please describe)

* 7. Your estimate for question 6 about AL methods used in CME activities in your CME unit is based on...

- ◯ Actual data
- ◯ Your overall perception (i.e., gut feel)
- ◯ Frequent attendance at lectures/courses/conferences
- ◯ Good-faith estimate

## Active Learning in Continuing Medical Education

### What are Your Beliefs about Active Learning?

* 8. Please tell us how much you agree or disagree with the following statements about AL.

| | Strongly Agree | Agree | Neither Agree nor Disagree | Disagree | Strongly Disagree |
|---|---|---|---|---|---|
| AL combines engagement and observation with reflection | ◯ | ◯ | ◯ | ◯ | ◯ |
| Both the instructor and the learners work cooperatively in AL | ◯ | ◯ | ◯ | ◯ | ◯ |
| Lectures (passive learning) are difficult to adapt to AL methods | ◯ | ◯ | ◯ | ◯ | ◯ |
| AL changes the teacher-learner relationship to a learner-learner relationship | ◯ | ◯ | ◯ | ◯ | ◯ |
| The learner is engaged so that both knowledge gained and recall are increased in AL | ◯ | ◯ | ◯ | ◯ | ◯ |
| With AL, instructors are more concerned with eliciting reflective thoughts that apply knowledge to practice than merely conveying facts | ◯ | ◯ | ◯ | ◯ | ◯ |

## Active Learning in Continuing Medical Education

### Barriers to Active Learning Methods

**Next, we'd like to explore barriers to using active learning methods.**

* 9. Each of the factors below may be perceived barriers for using AL in CME activities certified by your CME unit. Please indicate how much you agree or disagree with each of the following examples of perceived barriers.

| | Strongly agree | Agree | Neither Agree nor Disagree | Disagree | Strongly Disagree |
|---|---|---|---|---|---|
| AL is incompatible with larger audiences | ◯ | ◯ | ◯ | ◯ | ◯ |
| Many lecturers / presenters are unfamiliar with AL teaching methods | ◯ | ◯ | ◯ | ◯ | ◯ |

| | Strongly agree | Agree | Neither Agree nor Disagree | Disagree | Strongly Disagree |
|---|---|---|---|---|---|
| Learners dislike AL because it requires their participation | ○ | ○ | ○ | ○ | ○ |
| AL is an inefficient way of teaching because increased interaction with the audience is time-consuming | ○ | ○ | ○ | ○ | ○ |
| AL requires more preparation and planning as it is an unfamiliar teaching format for many presenters | ○ | ○ | ○ | ○ | ○ |
| Interaction with the audience may expose knowledge gaps of the lecturer/presenter | ○ | ○ | ○ | ○ | ○ |
| At my institution, AL is not considered a useful teaching tool in CME | ○ | ○ | ○ | ○ | ○ |
| At my institution, there is a lack of administrative / secretarial support for AL | ○ | ○ | ○ | ○ | ○ |
| My institution does not offer faculty development opportunities for AL teaching methods | ○ | ○ | ○ | ○ | ○ |
| Most presenters "grew-up" and studied in a predominately lecture-based system and it worked for them; so, they don't see a need to change | ○ | ○ | ○ | ○ | ○ |
| The culture of my institution is lecture-based | ○ | ○ | ○ | ○ | ○ |

If there are other barriers please describe them.

* 10. Please indicate how helpful you consider the following resources for increasing the use of AL methods in CME activities certified by your CME unit.

| | Very helpful | Moderately helpful | Somewhat helpful | A little helpful | Not at all helpful |
|---|---|---|---|---|---|
| Assistance from educational strategists | ○ | ○ | ○ | ○ | ○ |
| Institution-wide training sessions on AL | ○ | ○ | ○ | ○ | ○ |
| AL-related faculty development materials | ○ | ○ | ○ | ○ | ○ |
| Best-practice publications about AL | ○ | ○ | ○ | ○ | ○ |
| Institutional recognition for AL teaching / curriculum development | ○ | ○ | ○ | ○ | ○ |

If you have other ideas about increasing the use of active learning methods please describe them.

11. If you are developing best practices for increasing use of AL at your institution, please tell us about them.

## Active Learning in Continuing Medical Education

### Demographics

**Finally, we'd like to know a little more about you and your program/institution.**

* 12. Please provide the name of your program/institution. (This information will be kept confidential.)

* 13. What is your current position in your CME unit?

- ◯ Assistant/Associate Dean of CME
- ◯ Other Education Senior Leader (e.g., Senior Associate Dean of Education)
- ◯ CME Unit Director
- ◯ CME Unit Administrator
- ◯ CME Unit Coordinator
- ◯ CME Program Manager
- ◯ Other (please specify)

____________________

* 14. Who leads the CME unit in your program/institution?

- ◯ Same individual identified in question 13
- ◯ Other (please specify)

____________________

* 15. How many years has the CME unit leader been in that position?

- ◯ <1 year
- ◯ 1-3 years
- ◯ >3-6 years
- ◯ >6-9 years
- ◯ >9 years
- ◯ I prefer not to answer.

* 16. Is your CME unit accredited by the ACCME?

- ◯ Yes, **without** commendation
- ◯ Yes, **with** commendation
- ◯ No

* 17. How many CME activities (directly + jointly provided) on average do you report annually to the ACCME Program and Activity Reporting System (PARS)?

____________________

* 18. Is your CME unit based in a medical school?

- ◯ Yes
- ◯ No

## Active Learning in Continuing Medical Education

### Demographics

* 19. How many health-sciences faculty (basic science + clinical) are on staff in your medical school? Use this link to determine the exact number: AAMC Faculty Roster 2018.

[ ]

* 20. What is your gender?

- ◯ Male
- ◯ Female
- ◯ I prefer not to answer.

* 21. What is your age?

- ◯ 25-34
- ◯ 35-44
- ◯ 45-54
- ◯ 55-64
- ◯ 65+
- ◯ I prefer not to answer.

22. Please use the space below for any comments you may have.

[ ]

Thank you for taking the time to complete the questionnaire!
